# Potential Combinational Anti-Cancer Therapy in Non-Small Cell Lung Cancer with Traditional Chinese Medicine Sun-Bai-Pi Extract and Cisplatin

**DOI:** 10.1371/journal.pone.0155469

**Published:** 2016-05-12

**Authors:** Chia-Yi Tseng, Chin-Hung Lin, Lung-Yuan Wu, Jhih-Syuan Wang, Meng-Chi Chung, Jing-Fen Chang, Ming-Wei Chao

**Affiliations:** 1 Department of Biomedical Engineering, College of Engineering, Chung Yuan Christian University, Zhongli district, Taoyuan city, Taiwan; 2 Department of Bioscience Technology, College of Science, Chung Yuan Christian University, Zhongli district, Taoyuan city, Taiwan; 3 School of Chinese Medicine for Post-Baccalaureate, I-Shou University, Dashu District, Kaohsiung, Taiwan; National Cheng Kung University, TAIWAN

## Abstract

Traditional lung cancer treatments involve chemical or radiation therapies after surgical tumor removal; however, these procedures often kill normal cells as well. Recent studies indicate that chemotherapies, when combined with Traditional Chinese Medicines, may offer a new way to treat cancer. *In vitro* tests measuring the induction of autophagy and/or apoptosis were used to examine the cytotoxicity of SBPE, commonly used for lung inflammation on A549 cell line. The results indicated that intercellular levels of p62 and Atg12 were increased, LC3-I was cleaved into LC3-II, and autophagy was induced with SBPE only. After 24 hours, the apoptotic mechanism was induced. If the Cisplatin was added after cells reached the autophagy state, we observed synergistic effects of the two could achieve sufficient death of lung cancer cells. Therefore, the Cisplatin dosage used to induce apoptosis could be reduced by half, and the amount of time needed to achieve the inhibitory concentration of 50% was also half that of the original. In addition to inducing autophagy within a shortened period of time, the SBPE and chemotherapy drug combination therapy was able to achieve the objective of rapid low-dosage cancer cell elimination. Besides, SBPE was applied with Gemcitabine or Paclitaxel, and found that the combination treatment indeed achieve improved lung cancer cell killing effects. However, SBPE may also be less toxic to normal cells.

## Introduction

In Taiwan, approximately 10,000 new lung cancer cases occur each year, and 7000 people die from lung cancer annually [1], which is greater than those with colorectal, cervical, breast, prostate, and stomach cancers combined. These numbers continue to grow rapidly each year. There are numerous causes of lung cancer, and early symptoms are not always obvious. Lung cancer patients are often not aware of the early symptoms and miss opportunities for early diagnosis and treatment [2]. According to the Department of Health statistics, secondhand smoke, hot tar fumes, radiations, asbestos, factory smoke, soot, fine suspended particles, and dust storms are the primary causes of lung cancer [3–14]. Lung cancers are classified as small cell or non-small cell carcinomas according to whether they are non-epithelial or epithelium-derived, respectively [15]. Small cell carcinomas are highly malignant and can easily metastasize, especially if the cell-size is extremely small [16]. Therefore, chemical treatment is the preferred course of treatment for small cell carcinoma [17–19]. Lateral cases can be divided into squamous cell carcinoma, adenocarcinoma (including bronchioloalveolar carcinoma, also referred to as alveolar carcinoma), large cell carcinoma, glandular squamous cell carcinoma, carcinoid tumors, bronchial adenocarcinoma (including adenoid cystic carcinoma or mucinous epithelial carcinoma), etc [15, 20, 21]. Treatments for these types of cancers primarily involve surgical excision supplemented by radiation and chemotherapy [22, 23].

For treatment of conventional non-small cell lung cancer after surgical excision, chemotherapy kills normal cells along with the cancerous ones. The longer the chemotherapy administration continues, the stronger resistance that is developed by cancerous cells [24, 25]. Although this treatment method may provide the desired outcome, it also increases the risk for concurrent diseases [25]. Higher doses of chemotherapy drugs are needed during the terminal stages of cancers in order to achieve the same effects of lower doses administered during the earlier disease stages [20]. The side effects of the traditional treatment methods make them more difficult and less suitable for patients with more advanced stages of cancer or poorer health [26–29]. Based on the side effects and harm caused by these therapies, recent studies focused on the tumor cells and paid more attention to cellular immunotherapy, gene therapy, target drug therapy, etc [30–34]. Some studies tried to apply Chinese herbal medicines to cancer treatment [35–38]. These studies indicated that numerous Chinese herbal medicines, such as Chinese yew, Thalictrum fortune, Plumbagin, or Ganoderma lucidum [39–42], were found to reduce abnormal inflammation [43–45] and rapidly induce tumor cell apoptosis [46–48].

Sun-Bai-Pi (SBP) is the root bark of Morus alba L. According to the Encyclopedia of Traditional Chinese Medicine “Compendium of Materia Medica,” SBP is a key medicine used to remove water vapor from the lungs and to treat spitting blood, heated thirst, edema, fullness of the abdomen, bloating, urinary track problems, asthenic headache, internal energy deficiency, coughing, inflammation, diabetes, cancer, hepatitis, and heart diseases [49]. Previous studies indicated that the key ingredients of Sun-Bai Pi Extract (SBPE) included Morusin, Prenylflavonoid, and Benzofuran [50–53]. These are antioxidants that can reduce the NF-κB activity in cancer cells, cause cytotoxicity, and inhibit cancer metastasis, but their mechanism of action still remains unclear [54–57]. Therefore, in addition to investigating the mechanisms of SBPE-induced cancer cell death, we also need to establish a suitable synergistic cancer therapy that combines Chinese herbal medicines and chemotherapy drugs. In this study, we discovered that in addition to long-term apoptosis induction, SBPE also induced autophagy in A549 lung cancer cells. When the chemotherapy drug, Cisplatin, was added to the autophagic A549 cells, the results indicated that SBPE improved the cancer cell killing efficiency of chemotherapy drugs even at reduced doses.

## Materials and Methods

### Chemical

SBP collected from Di Lian Sheng Pharmaceuticals (DLSP, Taiwan), which was extracted by hot water for 30 min followed by centrifugtion, reserve dialysis and protein depletion. The pellet was discarded, the supernatant was collected and stored at 4°C. Cisplatin, gemcitabine, paclitaxel were purchased from Sigma-Aldrich.

### Cell culture

The human lung cancer alveolar A549 cells and normal lung fibroblast WI 38VA13 Subline 2RA cells were obtained from Bioresource Collection and Research Centre (BCRC, Taiwan). Cells were cultured in Ham’s F-12K medium (Sigma-Aldrich, St. Louis, MO, USA) with 10% fetal bovine serum (FBS, Gibco) at 37°C in a humidified atmosphere containing 5% CO_2_. For the experiments, the cells were treated with SBPE for several time points or treated with SBPE for 6 h followed by the additional chemotherapy drugs exposure for additional time points.

### MTS cytotoxicity assays

Viability was detected using a commercial MTS assay purchased from Promega (Madison, WI), measuring mitochondrial succinate dehydrogenase activity via conversion of MTS and phenazinemethosulfate to formazan. After treatment, a mixture of 10 μL water-soluble kit reagent plus 190 μL fresh medium was added to each well for a 1 h incubation at 37°C in the dark. Supernatants (100 μL/well) were collected, and the absorbance of the generated formazan was measured at 490 nm with a microreader.

### Autophagy immunofluorescence

After fixation and permeabilization, nonspecific reactivity was blocked by addition of 2% normal goat serum with 0.02% NaN_3_. The A549 cells were then incubated with primary anti-LC3 (Abcam) and anti-LAMP-1 (Abcam) monoclonal antibodies at a 1:200 dilution for 24 h, 4°C. Secondary antibody labeled with Alexa Fluor 488 (Invitrogen) or Alexa Fluor 594 (Invitrogen) was used and slides were covered with Prolong Gold (Invitrogen) anti-fade mounting media before usage. All images were observed on an Olympus IX51 microscope.

### Measurement of caspase 3 activity

Caspase activity was assessed with using the Caspase-Glo 3/7 assay kit (Promega). After SBPE treatment, the culture plates were removed from the incubator and allowed to equilibrate to room temperature for approximately 30 min, then the Caspase-Glo 3/7 assay reagent (200 μL/well) was applied according to the manufacturer’s instructions. Relative Luminance Unit (RLU) emitted by the product was measured using a microreader.

### Western blot

Proteins (40 μg/well/sample) separated electrophorectically on SDS gels were transferred to nitrocellulose membranes. Nonspecific reactivity of the membranes was blocked, and primary anti-p62 (Abcam), anti-Atg-12 (Cell signaling), anti-p53 (Abcam), anti-LC3 (Abcam), anti-Bcl-2 (Cell signaling), anti-Bax (Cell signaling), anti-Bad (Cell signaling), anti- α-tubulin (Abcam) were probed with appropriate dilutions. Secondary goat anti-mouse IgG or anti-rabbit IgG conjugated with HRP were used, and the blots were visualized by enhanced chemiluminescence (Promega™ ECL Western Blotting Substrate) and analyzed on the Odyssey Infrared imaging system (LI-COR Biosciences) (Lincoln, NE).

### Cell cycle analysis

After the treatments, the cells were collected and fixed in ice-cold 75% ethanol overnight at −20°C. The cells were subsequently centrifuged at 300 x g for 5 min and incubated with a propidium iodide (PI) working solution (100 μg/mL PI and 100 μg/mL RNaseA) for 30 min at 37°C. Cell cycle distribution was analyzed using a FACScan flow cytometer and analyzed with using ModFit software (Becton Dickinson, CA).

### Statistics

For statistical analysis, each experiment was performed in triplicate and repeated three times. The results were expressed as means ± SD for three independent experiments, and the differences between the groups were analyzed using Student t-tests with GraphPad statistics software. *, **, and *** indicate p<0.05, p<0.01, and p<0.001 significant difference, respectively, compared with the negative control. # and ## show p<0.05 and p<0.01, respectively, compared with samples with or without SBPE-treatment.

## Results

### Cell Viability

**Fig 1:**
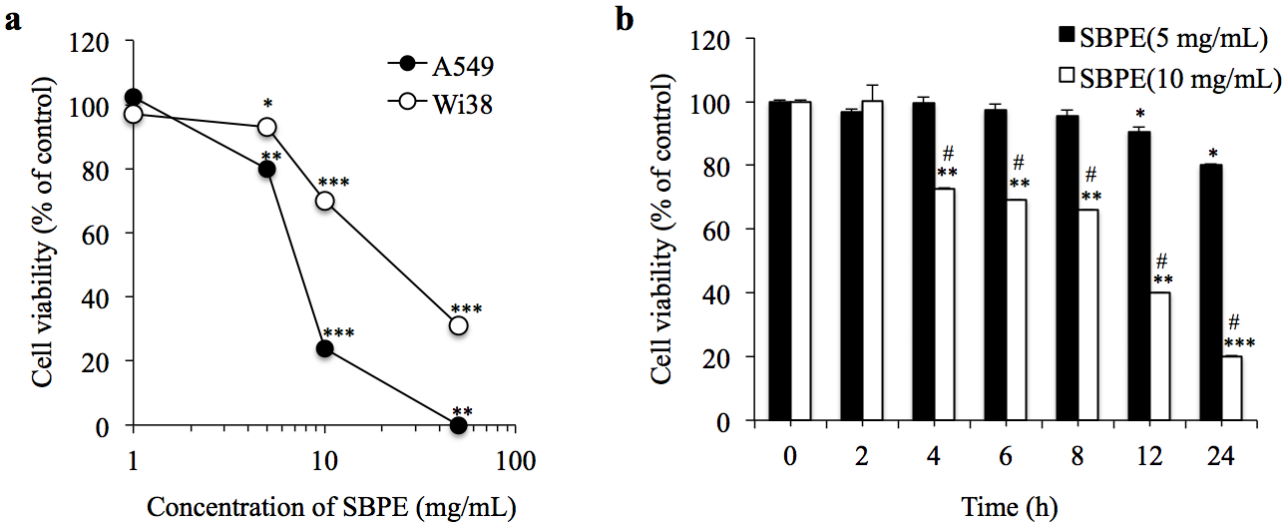
Cell viability. (a) Performed cell viability tests by administering different concentrations of SBPE to WI38 and A549 cells. (b) Administered 5 mg/mL and 10 mg/mL of SBPE to A549 and incubated the cells for various time points. Then, we used the MTS reagent to test their viabilities.

We used the MTS test to detect the effects SBPE had on the viability of WI 38VA13 Subline 2RA cells, normal lung fibroblast cells, and A549 lung cancer cells. As shown in Fig 1a, we found that 24 hours after administering SBPE, the vitality of the A549 cells was reduced as the SBPE dosage increased. The cell viability after administering 5 mg/mL was 85%, the IC50 was achieved after administering 9 mg/mL, and the cell viability after administering 10 mg/mL was 23%. On the contrary, SBPE showed no significant toxicity toward normal WI38 cells at the lowest dose tested, but significant cell death occurred within 24 hours following the treatment with SBPE at 5 and 10 mg/mL. If SBPE doses of 5 mg/mL and 10 mg/mL were used at different time points, we found that SBPE caused significant cell death within 24 hours.

### SBPE -induced Cancer Cell Autophagy Effect

**Fig 2:**
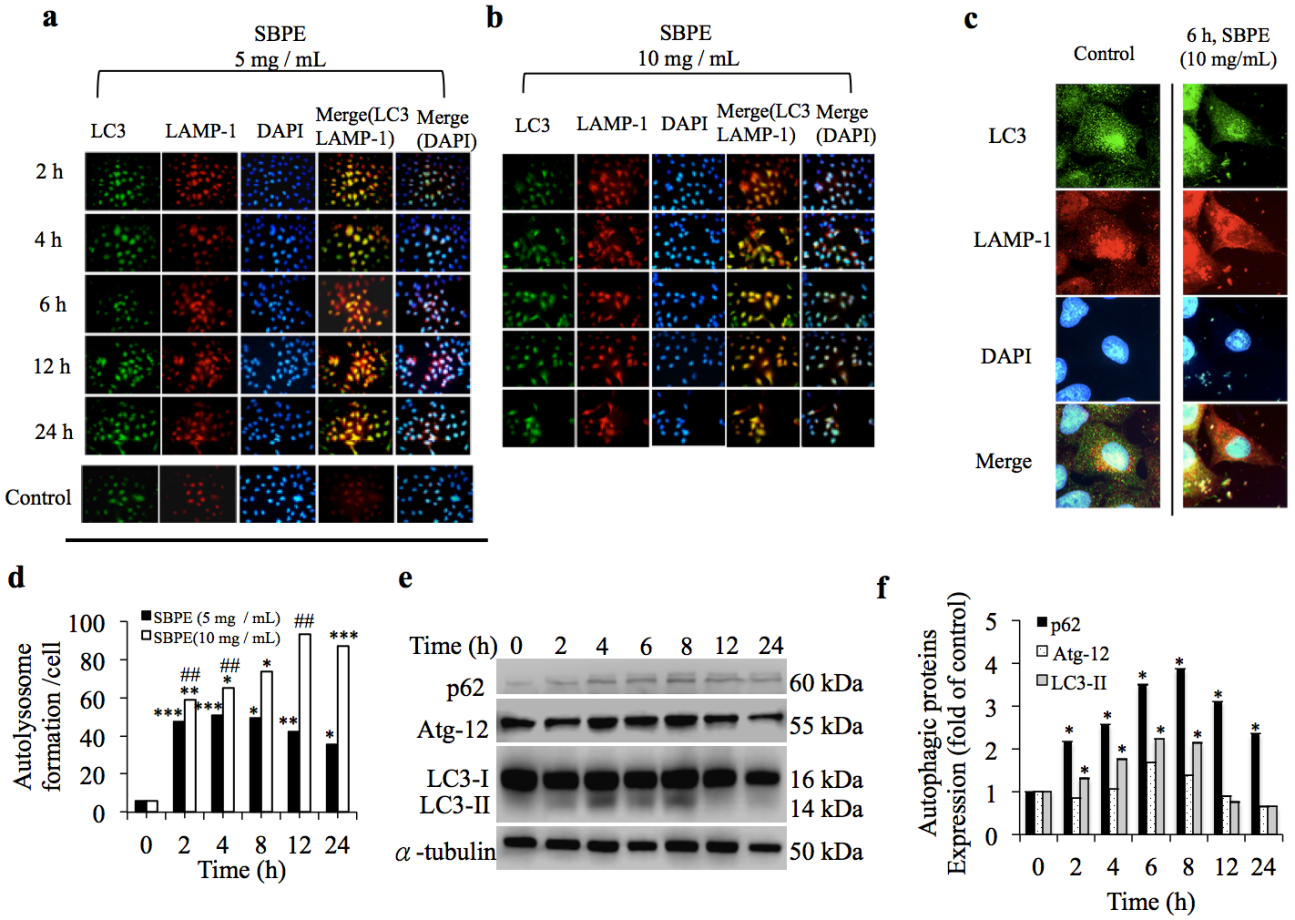
SBPE induced cancer cell autophagy. SBPE 5 mg/mL (a) and 10 mg/mL (b) were respectively added the cells and incubated for 2, 4, 6, 12, and 24 hours. Immunostaining was used to label the autophagy protein LC3 (green), LAMP-1 (red), and DAPI (blue) cancer cells. Magnification was 400X. (c) Magnified the cells incubated for six hours at 630X to observe the autophagy distribution. (d) Overlay of the green fluorescence (LC3) and the red fluorescence (LAMP-1), and calculated the number of cells with autophagic lysosomes. The vertical axis represents the total cell number per unit area divided by the number of cells with autophagic lysosomes. (e) Western blots were used to observe the effects SBPE had on the regulation of A549 cell autophagy related proteins, and found that the SBPE induced cell autophagy, which increased over time and peaked at eight hours. All the gels have been run under the same experimental conditions. (f) Statistical analyses for proteins performances.

To assess the mechanism, we assume that prior to the cell death, SBPE induces the cancer cells to become autophagic. As shown in Fig 2a and 2b, fluorescence microscopy observations indicated that regardless of the SBPE dosage. When the two fluorescent colors were superimposed and quantified, the autolysosome contents gradually increased over time. Therefore, the ratio of autolysosome formation for cells administered with SBPE 10 mg/mL significantly increased as a function of SBPE dose (Fig 2b). After ratio amplification, the autophagosome and lysosome overlaped, indicating that SBPE can indeed simulate A549 cells to become autophagic (Fig 2c). The statistical diagram shown in Fig 2d indicated that within 8 hours of SBPE (10 mg/mL) treatment, 70% of A549 cells generated lysosomal autophagy, and that rate increased to 90% in 12 hours. Western blots (Fig 2e) indicated that the p62 protein significantly increased four times that of the control group (Fig 2f) over 8 hours, and LC3-I was cleaved into LC3-II during this period. These results mean that the SBPE treatment induced the cancer cells to become autophagic, which peaked within eight hours. In addition, with using autophagy inhibitor, chloroquine (CQ) plus SBPE (10 mg/mL), we found the cell viability was significantly restored at 6 h and 24 h after treatment (S1 Fig).

### SBPE Induced Apoptosis

**Fig 3:**
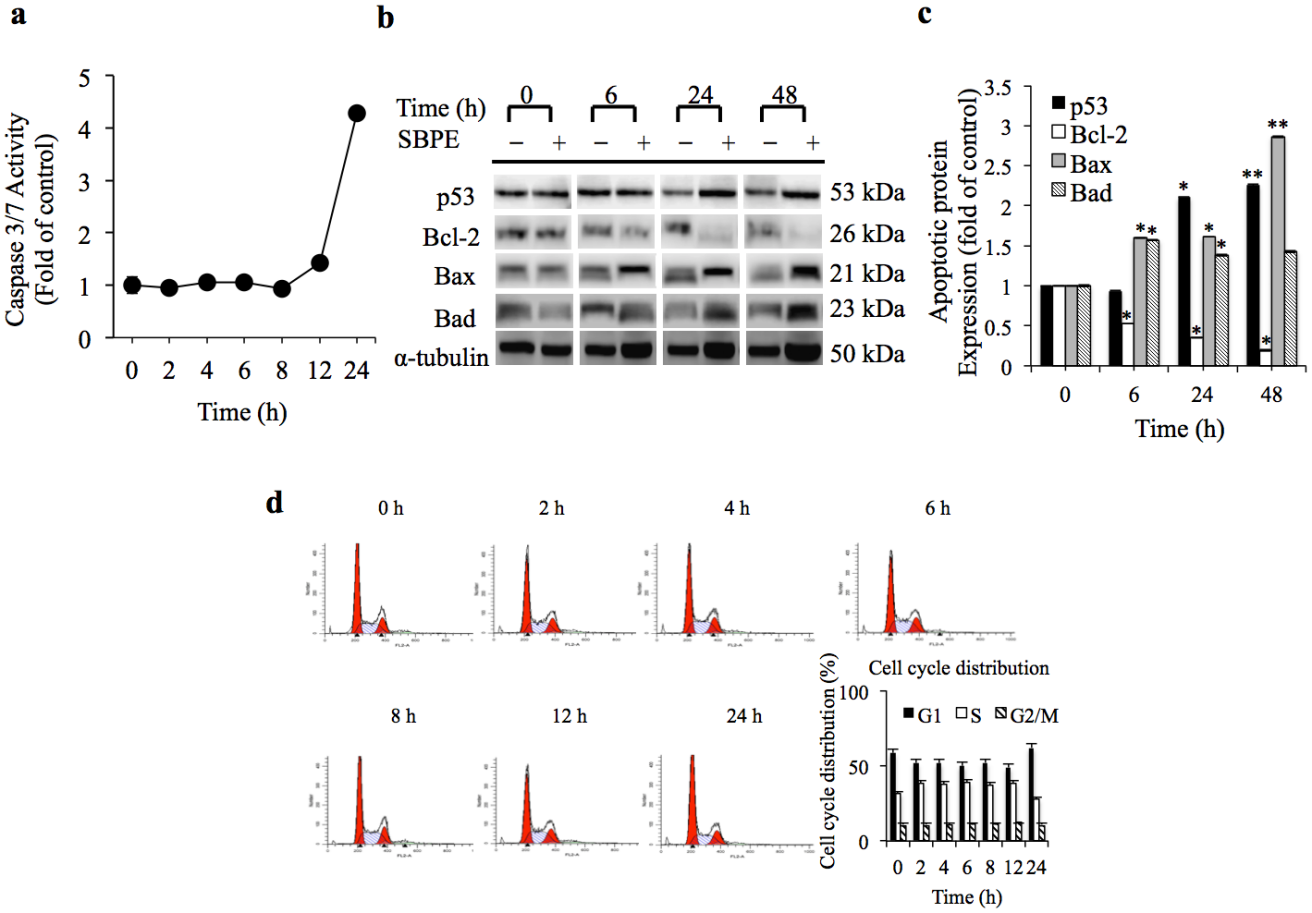
SBPE induced apoptosis. (a) After administration of SBPE 10 mg/mL; the Caspase3/7 reagent was used to detect their performances. (b) The effects that SBPE has on the protein control factors related to A549 cell apoptosis and anti-apoptosis. All the gels have been run under the same experimental conditions. (c) Statistical chart for protein performance. (d) FACS analyzes for cell cycle distribution, and use of the ModFit LT software to analyze cell cycle distribution.

SBPE was cytotoxic and the rate of autophagy induction strengthened as the dosage and administration time increased. However, it remains unknown whether administration of SBPE more than 12 hours can induce tumor cells to become apoptotic. Therefore, the apoptotic marker protein Caspase3/7 was detected to determine whether it is generated over time. Fig 3a indicates that the performance capacity of Caspase3/7 within A549 cells did amplify as the SBPE administration time increased. However, the amplification started at 12 hours and reached the peak performance capacity at 24 hours. Based on the forgoing results, SBPE can indeed initiate the apoptosis mechanism after 12 hours and can promote tumor cell autophagy within eight hours. Western blots (Fig 3b) indicated that the manifestations of the pro-apoptotic proteins p53, Bax, and Bad increased over time, while the anti-apoptotic protein Bcl-2 significantly decreased. According to Fig 3d, after the A549 lung cancer cells were treated with SBPE (10 mg/mL) for different periods of time, a flow cytometer was used to observe the cell distribution status during the cell cycle. The results indicated that as exposure time increased, the number of cells remaining in the G1 stage tended to increase, and the ratio of the S phase cells increased as time progressed, but declining after reaching the peak at 12 hours.

### Cisplatin and SBPE Combination Therapy can Reduce Cancer Cell Viability

**Fig 4:**
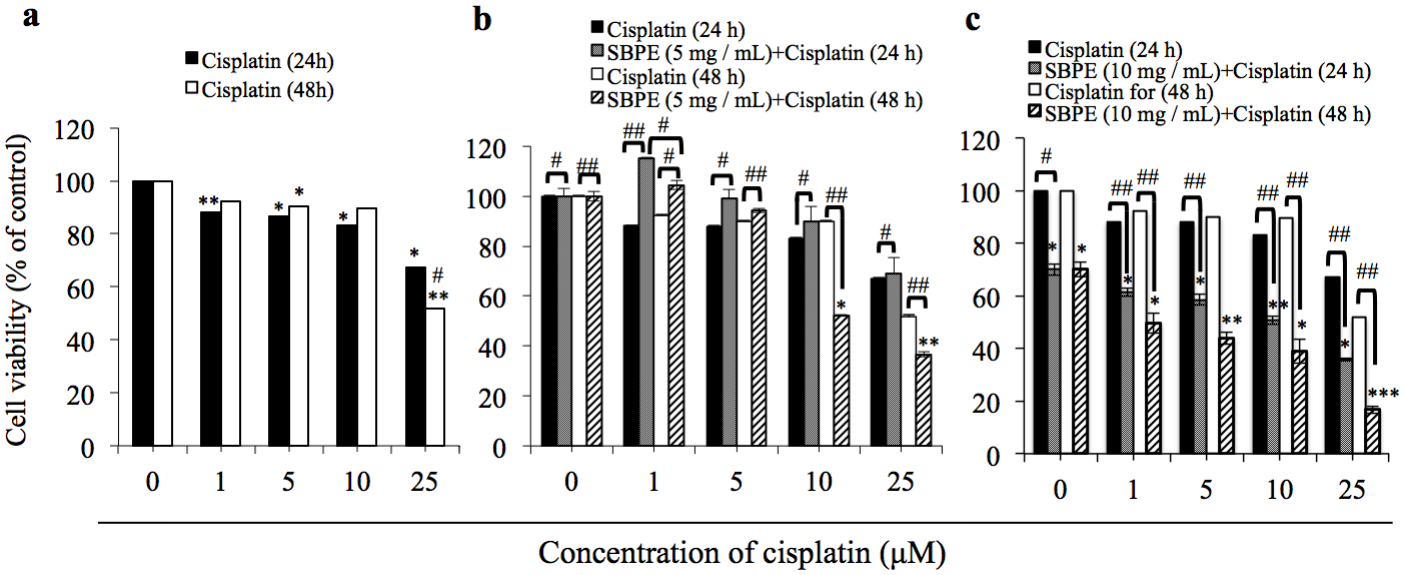
Cisplatin and SBPE combination therapy can reduce cancer cell survival rates. A549 tumor cell survival rate analyses after 1 μM, 5 μM, 10 μM, and 25 μM of Cisplatin was administered and incubated for 24 and 48 hours. The MTS reagent was used to detect the survival rates. Pre-treatment with using SBPE 5 mg/mL (a) and SBPE 10 mg/mL (b) doses of Cisplatin was added and incubated for 24 and 48 hours. The MTS reagent was used to detect the survival rates.

According to Fig 4, between 24 and 48 hours after Cisplatin was administered to A549 lung cancer cells, its cytotoxic effects strengthened as the Cisplatin concentration increased. However, the effects could only reach IC50 48 hours after 25 μM of Cisplatin was administered, and the treatment could only cause 33% cell death at the 24 hours mark. Therefore, we adopted the combination therapy method whereby SBPE was administered for six hours and the Cisplatin was administered for 24 hours. The objective was to use the Cisplatin treatment to kill the cancer cells after cell autophagy has been stimulated in hopes that this combination method can significantly strengthen the cytotoxic effect while reducing the Cisplatin dosage. As shown in Fig 4a, by administering 10 μM and 25 μM of Cisplatin for 48 hours after the cells were pretreated with SBPE (5 mg/mL) for six hours, the cancer cell viability can be reduced by 52% and 37%, respectively. Compared with the results, we found that the cells pre-treated with SBPE only needed Cisplatin 10 μM at 48 hours to achieve IC50, which is the same performance that can be achieved by Cisplatin 25 μM alone at the same time point. If the Cisplatin concentration is increased to 25 μM, the tumor cell killing effects are improved. As shown in Fig 4b, if SBPE 10 mg/mL was added prior to Cisplatin treatment for 24 hours, we found that cancer cell viabilities after 5 μM, 10 μM, and 25 μM Cisplatin treatments can be reduced to 58%, 50%, and 36%, respectively. Cisplatin 25 μM alone can only achieve IC50 concentration at 48 hours in the absence of SBPE. The cytotoxic effects of Cisplatin 10 μM can achieve IC50 after treatment for 48 hours. The evidence indicated that the pre-treated SBPE can indeed significantly strengthen the cancer cell cytotoxic effects of Cisplatin while reducing the Cisplatin dosage or treatment time.

### Apoptosis Induced by the Cisplatin and SBPE Combination Therapy

**Fig 5:**
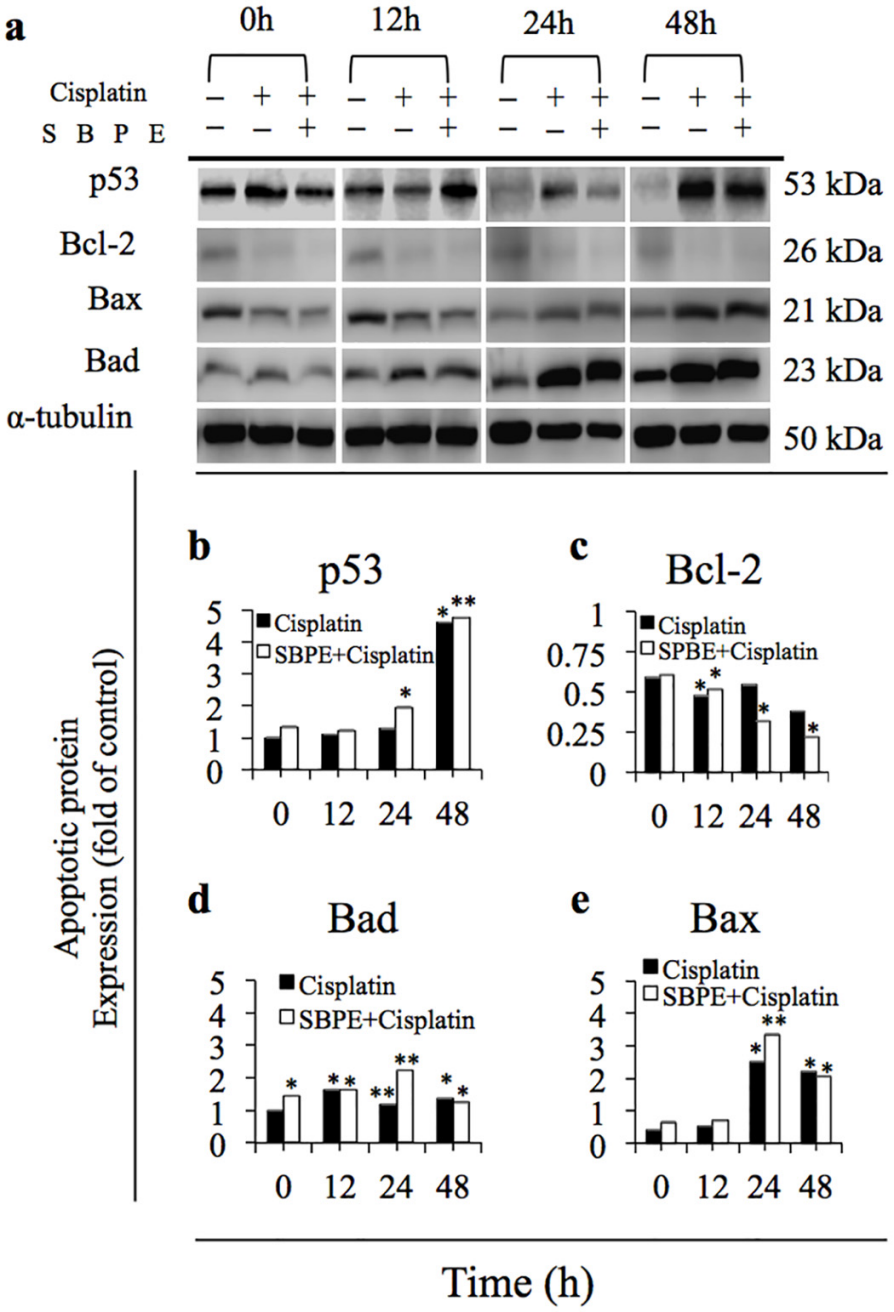
Apopthsis induced by the SBPE and Cisplatin combination therapy (a) 6 hours after treatment using 10 mg/mL of SBPE and 25 μM of Cisplatin was added and incubated for 24 and 48 hours. Western blots were used to observe the effects on the cell apoptosis phenomenon related proteins (p53, Bcl-2, Bax, and Bad). All the gels have been run under the same experimental conditions. Quantification chart for p53 (b); BCL-2 (c); Bax (d); Bad (e).

Western blots were used to observe how the combination therapy changed the regulation of apoptosis proteins (Fig 5a); and the results indicated that pro-apoptotic proteins p53 (Fig 5b), Bax (Fig 5d), and Bad (Fig 5e) increased as time progressed within 24 hours. This was especially true for p53, which significantly increased after only 12 hours. The anti-apoptotic protein, Bcl-2, significantly decreased as time progressed (Fig 5c) and was reduced by 32% after 24 hours, which achieved the effects one day earlier than the 37% reduction rate achieved after 48 hours when only Cisplatin was administered.

### Establishing an optimal SBPE Concentration for inducing Cancer Cell Cytotoxicity and an Optimal Time to administer additional Chemotherapy Drugs

**Fig 6:**
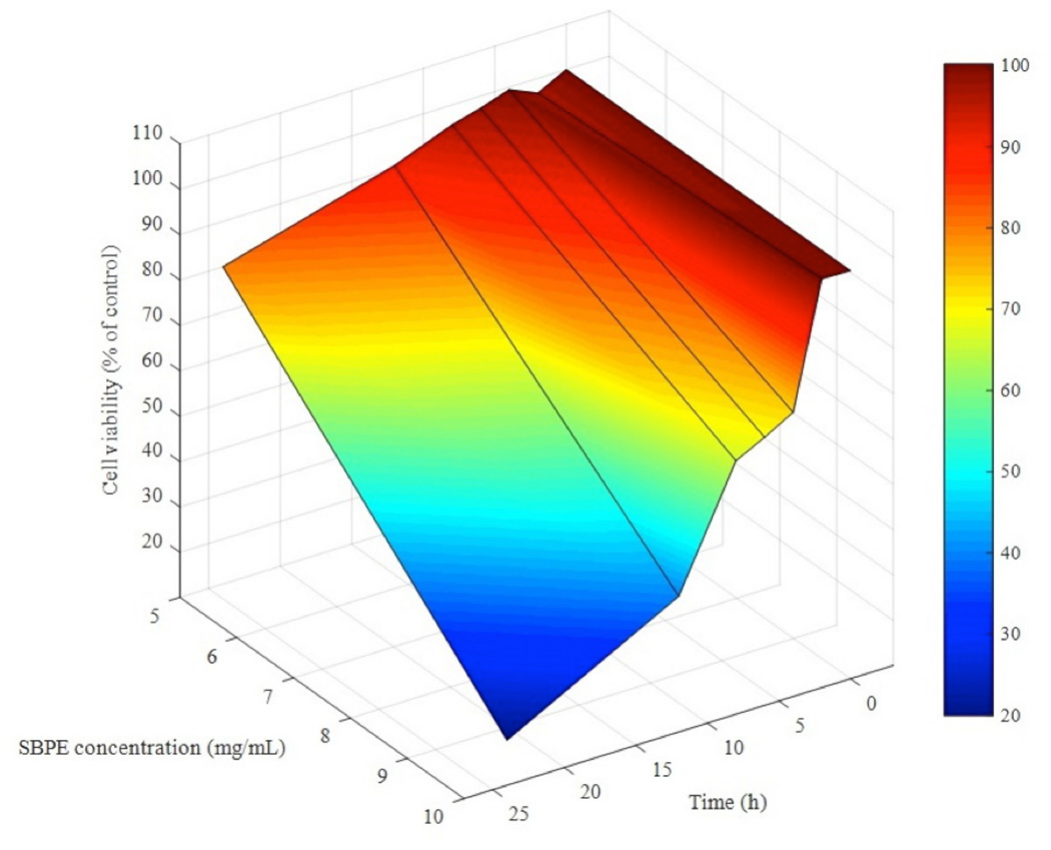
Optimal SBPE dosage against cancer cell survival rate. The MTS cytotoxicity test detection value was entered into the MATLAB mathematical analysis software through a statistical software to create a three-dimensional coordinate diagram on the relationship between concentration, time, and viability. XYZ were the variables. X-AXIS represents SBPE concentration X = [X, x1]; matrices X and x1 represent SBPE concentrations of 0 mg/mL, 1 mg/mL, 5 mg/mL, 10 mg/mL, 25 mg/mL, and 50 mg/mL; Y-AXIS represents SBPE action time Y = [Y, y1]; matrices Y and y1 represent 24 and 48 hours; Z-AXIS represents cell viability Z = [Z, z1]; and matrices Z and z1 represent viabilities after SBPE administration for 24 and 48 hours. The data matrices were inputted using surf commands and a three-dimensional surface diagram was plotted. The colors represent the action relationships between the three.

Our cytotoxicity analyses showed that as SBPE concentrations and treatment times increased so did their cytotoxic effect. Therefore, we established a relationship between A549 cancer cells, SBPE concentrations, and exposure times in order to optimally induce A549 cell autophagy (Fig 6). Using Matlab 2015a, we conducted a three-dimensional quantification of the existing (a) cell viability values; (b) SBPE concentrations; and (c) SBPE exposure times in order to reveal any relationships. According to Fig 2, we found that when the cell viability rate was at 70%, approximately 70% of the cells were induced to become autophagic. At this point, Cisplatin could then be added to accelerate the cytotoxic effects of the treatment. At a cell viability of 70%, we then picked ten time points at this dosage and derived a relational expression used to estimate the correlation between time and SBPE dosage. The relational expression for the time and dosage of SBPE on A549 lung cancer cell co-incubation was as follows:

$$y\;=\;0.0048x^{3}\;-\;0.1828x^{2}\;+\;1.6047x+\;5.7665$$

Where *y* represents the agent concentration given to the cells, *x* represents the implementation time, and 5.7665 is the calculation constant.

### Verifying the Optimal Dosage and Concentration for SBPE to Induce Cancer Cell Autophagy

**Fig 7:**
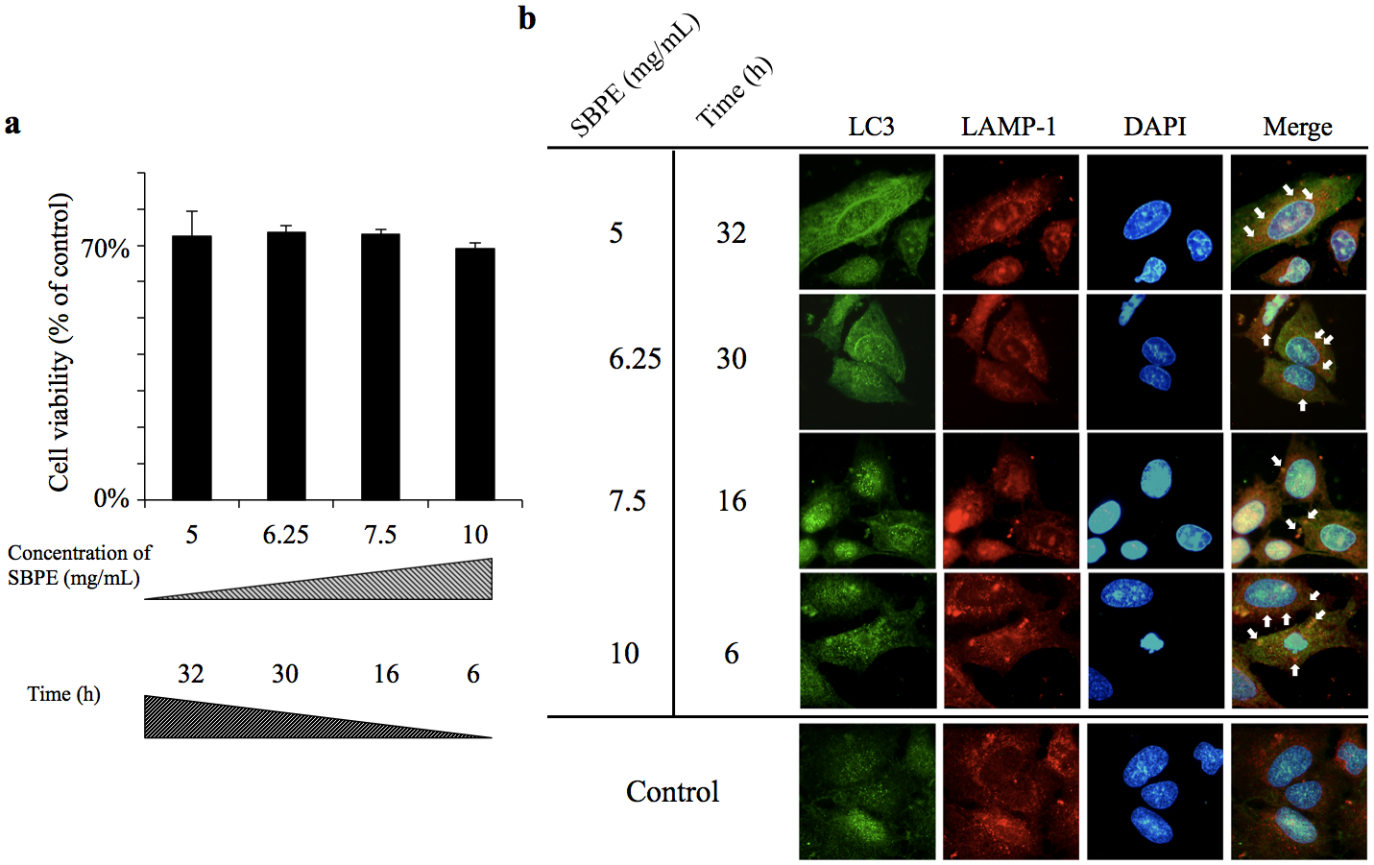
The actual effects that SBPE have on the optimal dosage against cancer cell viability. (a) Concentration and time verification against viability was conducted according to the mathematical analysis values generated by statistical software MATLAB as shown in Fig 6. SBPE concentrations of 5 mg/mL, 6.25 mg/mL, 7.5 mg/mL, and 10 mg/mL; timeframes were 32, 30, 16, and 6 hours; and MTS reagent was used to detect their activities. (b) Immunostaining was used to label the autophagy protein, LC3; lysosomal marker, LAMP-1; and nuclear marker, DAPI. The cells were incubated for eight hours and were magnified at 630X to observe the autophagy distribution. Arrows indicate autophagosomes (overlapped with green and red).

According to Fig 2, we found that when the cell viability rate was at 70%, approximately 70% of the cells were induced to become autophagic. Cisplatin could be added at this time to accelerate the cytotoxic effects. According to Fig 6 and the formula shown, for cells to achieve an autophagic state, the cells must be given SBPE for a certain period of time in order for them to achieve a 70% survival rate. We conducted MTS tests on SBPE 5 mg/mL (32 h), 6.25 mg/mL (30 h), 7.5 mg/mL (16 h), and 10 mg/mL (6 h) to verify that this timeframe provided the optimal dosage and concentration for SBPE to induce cancer cell autophagy. The results indicated that cell viability could be maintained at 70% as shown in Fig 7a. We performed fluorescence microscopy and found that LC3 overlapped with LAMP-1 when SBPE was administered at these 4 concentrations. This observation represented an overlap of the autophagosome and the lysosome. Therefore, administering SBPE for a specific period of time can indeed stimulate A549 cell autophagy (Fig 7b).

### The Effects that Combining SBPE to Chemotherapy Drugs Gemcitabine and Paclitaxel have on Cancer Cell Viability Rate

**Fig 8:**
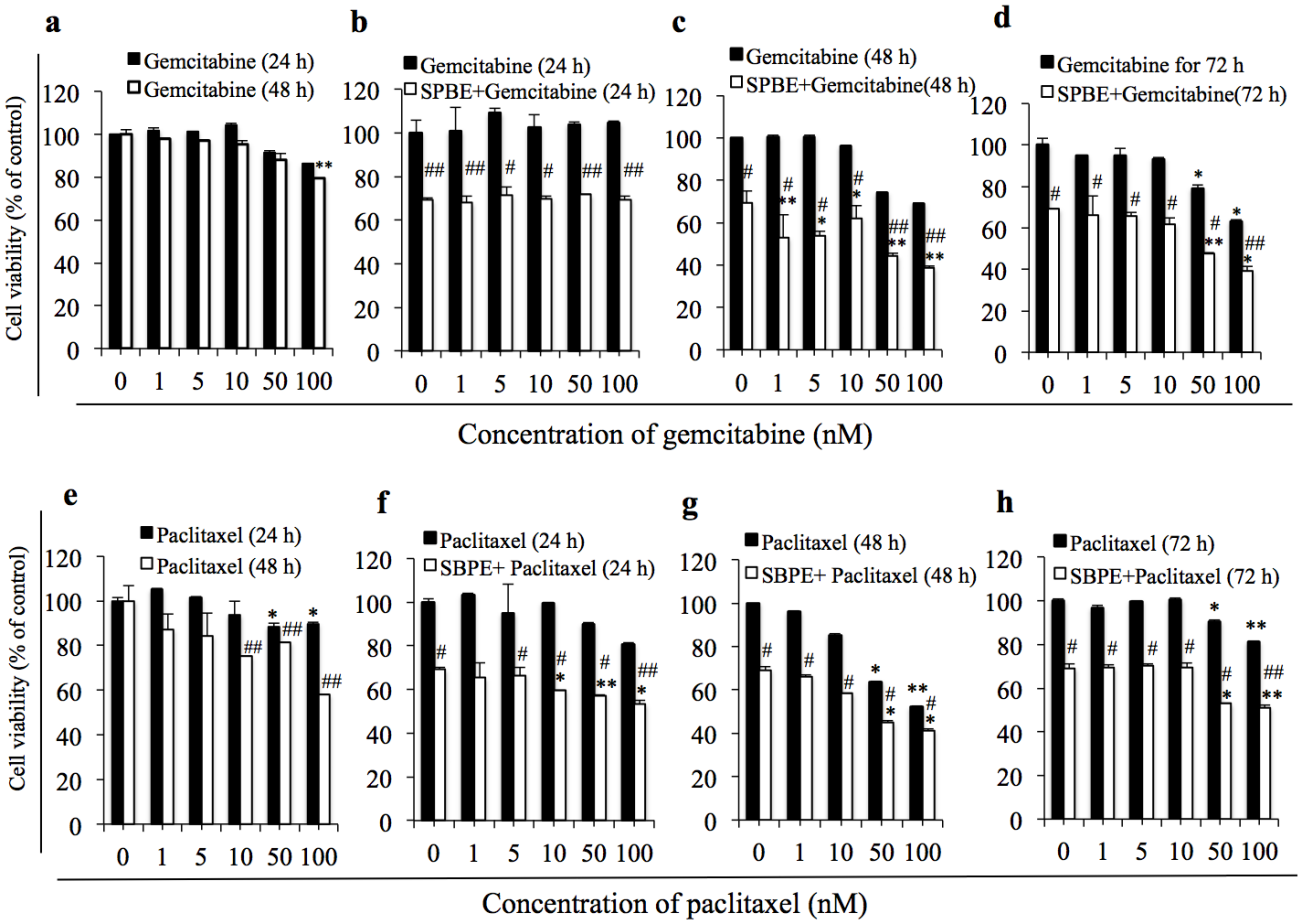
The effects that combining SBPE to chemotherapy drugs Gemcitabine and Paclitaxel have on cancer cell viability. (a) A549 tumor cell viability analysis after the administration of Gemcitabine. After 1 μM, 5 μM, 10 μM, 50 μM, and 100 μM of Gemcitabine was administered for 24 and 48 hours, the MTS reagent was used to detect their activities. Secondly, after 6 hours of treatment with 10 mg/mL of SBPE; Gemcitabine was added for 24 hours (b), 48 hours (c) and 72 hours (d). (e) A549 tumor cell viability analysis after treatment with 1 μM, 5 μM, 10 μM, 50 μM, and 100 μM of Paclitaxel and incubated for 24 and 48 hours. The MTS reagent was used to detect their activities. Secondly, after 6 hours of treatment with 10 mg/mL of SBPE; Paclitaxel treatment was added for 24 hours (f), 48 hours (g) and 72 hours (h).

According to Fig 8, the cytotoxic effects of Gemcitabine and Paclitaxel within 72 hours can be improved as the SBPE concentration is increased. However, Gemcitabine required administration of 100 μM for up to 72 hours and Paclitaxel required administration of 100 μM for up to 48 hours in order to achieve IC50. Similar to Cisplatin, we pre-treated the cells with SBPE for six hours and then added the Gemcitabine or Paclitaxel combination in order to observe whether SBPE can significantly increase the cytotoxic effects of Gemcitabine or Paclitaxel while also reducing their dosage. The results indicated that after treatment using 50 μM of Gemcitabine and Paclitaxel for 48 hours, the survival rates were effectively reduced from 78% to 44% and 65% to 45%, respectively. When compared to non-treated results (Fig 8a and 8e), the SBPE pre-treated cells only required half the amount of Gemcitabine or Paclitaxel in 48 hours to achieve IC50.

## Discussion

There are numerous side effects associated with existing traditional chemotherapy drugs [26–29]. For example, Cisplatin, a chemotherapy drug commonly used to treat lung cancer has a long response time, is prone to drug resistance, and long-term application may inhibit apoptosis or produce uncomfortable side effects to patients [58]. Another chemotherapy drug, Gemcitabine, may cause uncomfortable symptoms, such as dizziness, vomiting, leukopenia, thrombocytopenia, and kidney toxicity [59]. Traditional Chinese herbal medicines are beginning to emerge as anti-cancer drugs [35–38]. At present, approximately two-thirds of cancer patients are at the mid- to late-stage of their diagnosis because of diagnostic technique restrictions and reduced surgical opportunities [2, 15–23]. Patients during these advanced stages are too physically weak to withstand the trauma of surgery and the toxic side effects of chemotherapy. Conventional cancer surgery, radiation, and chemotherapy treatment methods all have limitations, such as the extent of the patients’ pathology, treatment time, age, physical condition, the existence of complications, and drug allergies. Some patients may be reluctant to accept these treatment methods or would stop treatment due to the side effects [2, 15–23]. Chinese herbal medicines lack these restrictions and can be used accordingly [60, 61]. The advantages of Chinese herbal medicines are more than merely the cytotoxic effects against cancer cells, they also enhance the benefits from physical fitness, induce immune cytokines, and strengthen cellular immunity; thereby, providing protection against cancer. However, there are no large-scale clinical trials using traditional Chinese medicines that repeatedly demonstrate their “evidence-based medical” efficacy. Therefore, Chinese herbal medicines are still being classified as secondary and alternative medicines for cancer treatment.

In this study, we investigated whether the traditional herbal medicine, SBPE, can induce tumor cell autophagy. We further administered Cisplatin to observe whether SBPE can kill lung cancer cells while also reducing Cisplatin dosages. Finally, we compared the effects produced by other chemotherapy drugs in hopes to establish a Chinese-Western combination therapy treatment. Our results indicated that SBPE would not produce significant cytotoxicity to normal lung epithelial cells but would cause lung cancer cell death under the same concentrations. We also found that lung cancer cells would reach their IC50 after treatment with 10 mg/mL SBPE for 24 hours. Although the mechanism of SBPE’s lower cytotoxicity toward normal cells remains unknown, these results mean that SBPE can efficiently kill lung cancer cells without harming normal cells.

When autophagy occurs, LC3-labeled autophagosomes form autophagic lysosomes with LAMP-1-labeled lysosomes. We found that SBPE could induce autophagy of A549 cells and this became more obvious as time progressed. The autophagic lysosomes in the treated cells reached a maximum volume eight hours after drug administration. Expression of the labeled, lysosomal protein, p62, and the labeled, autophagosomal protein, Atg12, verified our fluorescent data. Eight hours after drug administration, LC3-I cleaved the maximum amount of LC3-II. After 12 hours, lung cancer cells began to apoptosis, evidenced by increased protein expression of the apoptotic and pro-apoptotic markers and the reduced expression of the anti-apoptotic marker. Based on the results discussed above, SBPE can induce lung cancer cell apoptosis after autophagy. However, although the number of cells in G1 phase tended to increase after 24 hours during the cell cycle analysis, the number of cells in G2/M phase did not change and there was no sign showing that SBPE can cause cell cycle arrest for cancerous cells. Interestingly, the number of cells in S phase increased from 31% to 38% as the SPBE treatment time progressed, but dropped back to 30% after 24 hours. The cause of this effect is unknown, but it is estimated that most of the cells moved towards apoptosis and a small portion of the cells moved towards the G1 phase arrest after 24 hours.

According to Fig 4, it took 48 hours to reach IC50 following the treatment of A549 cells with the chemotherapy drug, Cisplatin. Further research found that pre-treating lung cancer cells with 10 mg/mL SBPE for six hours caused 30% of the treated cells to die and induced autophagy in 50% of those cells. Cancer cell survival rate tended to decrease in cells pre-treated with SBPE compared to groups that were not pre-treated. Furthermore, the Cisplatin concentration and treatment time required to reach IC50 was significantly reduced. The tendency chart in Fig 6 indicated that the greater the SBPE concentration and treatment time, the stronger the cytotoxic effects within 24 hours. According to our Matlab analysis chart, we found that when concentration levels reached the blue region in the chart, cytotoxicity was almost 80%. Treating with 5 mg/mL SBPE for 24 hours reduced cell viability by up to 80%. Treatment with 10 mg/mL SBPE for six hours reduced cell viability by up to 70%. The lowest viability rate of 20% was achieved after treating with 10 mg/mL SBPE for 24 hours. However, we found that the most appropriate time to administer Cisplatin was when the cells were maintained in the autophagy state (yellow region of the chart). As shown in Fig 7, we were able to significantly reduce cell viability and increase apoptosis rates of treated A549 cells. These results indicate that our SBPE pre-treatment and chemotherapy drug combination therapy produced better lung cancer cell elimination capabilities compared to administering the chemotherapy drug alone. To expand these results, we also combined SBPE with two other chemotherapy drugs, Gemcitabine and Paclitaxel, and found that our SBPE pre-treatment and chemotherapy drug combination can indeed achieve improved lung cancer cell killing effects.

In this study, we first established that pre-treatment with SBPE and the later addition of Cisplatin can induce lung cancer cell autophagy. We then combined SBPE with other existing chemotherapy drugs to establish a “Chinese-Western combination therapy” that is more efficacious, provides a shorter chemotherapy treatment course, and lowers chemotherapy drug dosage.

## Supporting Information

S1 Fig Performed cell viability tests by administering SBPE (10 mg/mL) plus or minus autophagy inhibitor, chloroquine (CQ, 10 μM) to A549 and incubated the cells for 0, 6, 24h. Then, we used the MTS reagent to test their viabilities.
(TIF)
