# Supplementary figures and images for: Potential Combinational Anti-Cancer Therapy in Non-Small Cell Lung Cancer with Traditional Chinese Medicine Sun-Bai-Pi Extract and Cisplatin
Source: PLoS One. 2016 May 12;11(5):e0155469. doi: 10.1371/journal.pone.0155469 (PMC4865219; doi:10.1371/journal.pone.0155469)

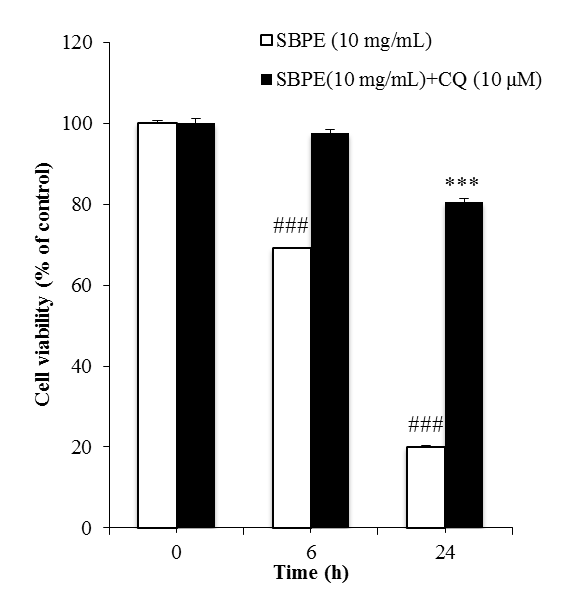

Supplement: S1 Fig — Then, we used the MTS reagent to test their viabilities. (TIF) [file pone.0155469.s001.tif]
